# Herpes Simplex Type 1 UL43 Multiple Membrane-Spanning Protein Increases Energy Metabolism in Host Cells through Interacting with ARL2

**DOI:** 10.3390/cells11223594

**Published:** 2022-11-14

**Authors:** Jianshan Deng, Zhiying Zhong, Chengxu Geng, Zhenning Dai, Weihan Zheng, Ziyue Li, Zi Yan, Jiaxin Yang, Wenfeng Deng, Wei Tan, Hanxiao Sun, Shiyu Li

**Affiliations:** 1 Institute of Genomic Medicine, College of Pharmacy, Jinan University, Guangzhou 511436, China; 2 Guangdong Medical Innovation Platform for Translation of 3D Printing Application, The Third Affiliated Hospital of Southern Medical University, Southern Medical University, Guangzhou 510630, China; 3 Department of Stomatology, Guangdong Second Traditional Chinese Medicine Hospital, Guangzhou 510095, China; 4 Department of Anatomy, Guangdong Provincial Key Laboratory of Digital Medicine and Biomechanics, School of Basic Medical Sciences, Southern Medical University, Guangzhou 510515, China; 5 Guangzhou Women and Children’s Medical Center, Guangzhou Medical University, Guangzhou 510799, China; 6 Guangxi Key Laboratory of Birth Defects Research and Prevention, Nanning 530005, China

**Keywords:** herpes simplex virus type 1, UL43 protein, ATP generation, small G protein, ANT1

## Abstract

Non-essential proteins for viral replication affect host cell metabolism, while the function of the UL43 protein of herpes simplex virus 1 (HSV-1) is not clear. Herein, we performed a comprehensive microarray analysis of HUVEC cells infected with HSV-1 and its UL43-deficient mutant and found significant variation in genes associated with cellular energy metabolic pathways. The localization of UL43 protein in host cells and how it affects cellular energy metabolism pathways were further investigated. Internalization analysis showed that the UL43 protein could be endocytosis-mediated by YPLF motif (aa144–147) and localized to mitochondria. At the same time, more ATP was produced by coupling with mitochondrial small G protein ARF-like 2 (ARL2) GTPase, which triggered the phosphorylation of ANT1 (SLC25A4) to affect the opening degree of mitochondrial permeability transition pore (mPTP), and significantly promoted the aerobic oxidation and oxidative phosphorylation of glucose. Our study shows that UL43 mediates the improvement of host cell metabolism after HSV-1 infection. Additionally, UL43 protein could be a valuable ATP-stimulating factor for mammalian cells.

## 1. Introduction

Herpes simplex virus 1 (HSV-1) is a ubiquitously found α-herpesvirus capable of producing productive and latent infections in the human host. Similar to all viruses, HSV-1 relies on the metabolic network of the host cell to provide energy, and macromolecular precursors to facilitate viral replication. HSV-1 is not only dependent on the metabolic activity of the underlying host cell, it also actively redirects the metabolism of the host cell which is tampered with [1].

HSV-1 encodes 80 genes; approximately half of these genes are not involved in viral replication under culture conditions [1,2]. Although the deletion of nonessential genes in cell culture will not inhibit viral replication, these genes are usually essential for replication in natural hosts, as mutant viruses with deletion of nonessential genes are rarely isolated from patients [2]. The HSV-1 gene UL46 encodes the phosphorylation protein VP11/12, and UL47 encodes the phosphorylation protein VP13/14, both of which interfere with the phosphorylation signaling pathway in host cells [3]. During HSV-1 infection in oligodendrocytes, UL46 also interacts with the small GTPase Rab27a, which reduces viral growth and infectivity in Rab27a-depleted cells [4,5]. UL46 was also recently found to activate mTORC1 in fibroblasts [6]. US3 was also found to affect the subcellular localization of certain viral proteins by influencing their phosphorylation status, which leads to a decrease in the amount of gB found on the cell surface by phosphorylating their cytoplasmic tail at Thr-887 and possibly enhancing their endocytosis [7]. ICP22, encoded by US1 of HSV-1, binds to the immune response to infection via the T-cell co-stimulatory molecule CD80 promoter, and ICP22 may also modulate the host E3 ubiquitin ligase against immune system action [8,9,10,11]. Increased susceptibility of UL41 mutant viruses to interferon-α and -β was also demonstrated in mature dendritic cells, and HSV-1 requires virion host shutoff protein (the protein encoding UL41) to block the phosphorylation of STAT1 and IFNγ signaling [12].

HSV-1 infection can affect mitochondrial function. UL7 may be an important protein in this process, and mitochondria may also display the function of UL7. UL7 has been identified as a partner of adenine nucleotide translocase (ANT2) by mass spectrometry and affinity purification techniques [13]. The known function of UL12.5 in HSV-1 appears to be related to mitochondrial stress, as mitochondrial DNA has been found to be eliminated in a UL12.5-dependent manner early in HSV-1 infection, a process which also involves mitochondrial nucleases [14,15].

Taken together, many non-essential proteins of HSV-1 act as components of the complex functional network of the host, such as UL11/gE/gD, UL16/gE/UL11/UL21/VP22, UL31/UL34/US3 or ICP0/UL46/UL47/ICP22/US3/UL13/VP22 [2], affecting the function of the host cell in order to execute the purpose of parasitism and plunder.

The UL43 gene-encoding protein of HSV-1, is not present in mature extracellular viral particles; much as in the replication of nonessential proteins described above, deletion of UL43 in mutant viral strains will not affect HSV-1 replication [16]. However, other functions of UL43 are still unknown. According to its sequence, the protein encoded by UL43 is a non-glycosylated transmembrane protein that may contain seven transmembrane domains consisting almost entirely of alpha helices [17]. The transmembrane proteins gB, gD, gH, and gC encoded by HSV-1 usually promote membrane fusion of the virus and host. UL43 is also a transmembrane protein, but our previous study found that the lack of UL43 expression did not affect the expression levels of the fusion proteins gB, gD, gH or/and gC [18]. Further, according to the sequence, a YDLF endocytosis motif is located in UL43, and so we speculated that UL43 would cause functional changes in host cells. We are interested in how UL43 enters host cells and elucidates its functional role in the host.

In this study, the function of UL43 protein was analyzed by gene chip of UL43 gene deletion mutant (HSV-1 △UL43) and normal virus HSV-1. The effect of UL43 protein domain YPLF motif (aa144-147) mutation on endocytosis and its mitochondrial localization. At the same time, the cell proteins interacting with UL43 were identified to further study the mechanism of UL43 on cell energy metabolism.

## 2. Materials and Methods

### 2.1. Cells and Viruses

Human umbilical vein endothelial (HUVEC) cells (YS806C, Shanghai Yaji Biotechnology Co., Ltd., Shanghai, China) were cultured and preserved in our laboratory and maintained in Dulbecco’s modified Eagle’s medium (Invitrogen, Carlsbad, CA, USA) containing 10% FBSF and supplemented with 100 units/mL penicillin and 100 μg/mL streptomycin in 0.3% collagen-coated dishes. The HSV-1 wild-type strain F (HSV-1) and E. coli DH5α-competent cell receptor bacteria were preserved in our laboratory. The HSV-1 and HSV-1 △UL43 mutants were reported elsewhere [17].

### 2.2. Plasmids and constructs

UL43 gene of HSV-1 ((NC-001806.2 from NCBI database) was cloned into pcDNA3.1 plasmid (Invitrogen, Carlsbad, CA, USA) with synthetic oligonucleotides UL43-for (5’- CCGAATTCAAGCTTATGCTCCGCAACGACAGCC) and UL43-rev (5’- CGCGGATCCTTTATTGAAAAATATATCAA), named pUL43. Mutated nucleotides (GCC and GGC) were inserted in the middle of the primer. The pUL43-Y144A mutant, carrying tyrosine (Y)-to-alanine (A) substitutions in the cytoplasmic tyrosine-based motifs, was constructed by using pUL43 plasmid. The oligonucleotides used were Y144A-s (5’-CTCGCCGATGACGTCgccGCCCCGCTCTTTCTCCTCGCCCCG-3’) and Y144A-as (5’-GAGGAGAAAGAGCGGggcGACGTCATCGGCGAGCAC-3’) (The mutated nucleotides are indicated in lowercase letters). To construct an expression vector pCMV(f)UL43-WT, whose UL43 gene was tagged with only the FLAG epitope sequence (DYKDDDDK), a UL43 ORF without a start codon was PCR amplified and inserted into the EcoRI and BamHI sites of pFLAG-CMV-2 (Sigma, St. Louis, MO, USA). Additionally, they constructed pCMV(f)UL43-Y144A. HUVEC cells transfected with pCMV(f)UL43-WT or pCMV(f)UL43-Y144A expressed the UL43-WT-FLAG and UL43-Y144A-FLAG, respectively. ARL2-siRNA vectors were constructed from pSINsi-mU6 DNA^TM^ (Takara Bio, Ohtsu, Japan). Double-stranded oligonucleotides were inserted into pSINsi-mU6DNA^TM^ at BamHI/ClaI sites. The oligonucleotides that targeted specific genes and scrambled control (scrambled siRNA) were as follows: ARL2 (5’-GGATTCAAGCTGAACATCT-3’) (ARL2-siRNA) and scrambled control (5’-AATAATAATGGGGGGATCC-3’) [19]. All mutants were verified by sequencing.

### 2.3. Determination of Virus Titer and Plaque

HUVEC cells in logarithmic growth phase were washed three times with PBS, digested with trypsin, centrifuged (1000 rpm, 5 min), discarded the supernatant, and added the cell maintenance medium (2% DMEM medium) to the cell concentration of 1 × 10^5^/mL, 100 μL per well, added to the 96-well plates. Dilute the virus with a cell maintenance solution, starting at a maximum dilution factor (1 × 10^9^), 100 μL per well. Cells were harvested within 24 h after infection with a multiplicity of infection (MOI) of 5, and progeny virus titers were determined. To determine plaque size and number, infected cells were incubated under a 2% agarose cover for 2 days. The infected cells were fixed with 2.5% formaldehyde and stained with 1% crystal violet in 50% ethanol. Plaques were measured using an inverted microscope (Nikon, Tokyo, Japan) and the average diameter was calculated. Virus titer (PFU/mL) = plaque number/dilution ratio/inoculation volume per well (ml), and MOI = (virus titer x virus volume)/cell number.

### 2.4. Microarray Analysis

HUVEC cells grown on cover slips were infected with HSV-1 and HSV-1 △UL43 at MOI of 5. After 24 h, the cells were lysed, and total RNA was isolated with Trizol (Invitrogen, Carlsbad, CA, USA) and purified with RNeasy Mini kit (Qiagen, Valencia, CA, USA). Three biological replicates were performed for each experimental condition. Samples preparation and hybridization according to the whole human genome gene expression microarray were performed at Kangcheng Bio-tech (Shanghai, China). Functional analyses of selected genes were carried out using Gene Ontology project (http://www.geneontology.org, accessed on 29 March 2021). Genes with a similar expressed tendency were classified by cluster analysis based on values of normalized expression (correlation > 0.99). Endeavour software (http://homes.esat.kuleuven.be, accessed on 29 March 2021) and ToppGene (http://toppgene.cchmc.org, accessed on 29 March 2021) [20,21] were used for prioritization based on the similarity of the known genes. Through network-based topological feature analysis, we selected specific genes (specificity = 80%) and sensitive genes (sensitivity = 90%) from all differentially expressed genes. The software Endeavor and ToppGene for functional consistency analysis can identify 85% and 91% of known related genes, respectively, and screen differentially expressed genes 2 times or more.

### 2.5. Quantitative Real-Time PCR

To validate microarray results, mRNA expression of selected genes was monitored using quantitative real-time PCR (qRT-PCR). Since the expression of β-actin was infected by HSV-1, β-Tubulin was selected as the internal reference. The genes were amplified using the respective primer, as described in Table S1. PCR products were separated on 2% agarose and stained with ethidium bromide. In addition to using qRT-PCR to analyze the mRNA expression of seven differentially expressed genes, the mRNA expression of four genes of mitochondrial NADH dehydrogenase (ubiquinone) 1 beta subcomplex subunit 3 (NDUFB3), succinate dehydrogenase subunit B (SDHB), cytochrome c-1 (CYC1) and surfeit locus protein 1 (SURF1) were also detected. The time PCR detection system (Bio-Rad Laboratories, Hercules, CA, USA) and Maxima SYBR Green qPCR Master Mix (2x) (Fermentas, Burlington, Ontario, Canada) were used.

### 2.6. Construction of Plasmid Transfected Cells

HUVEC cells were seeded on cover slips in 12-well dishes prior to the beginning of the experiment. When the cell density reached 80–90%, the empty plasmid, pCMV(f)UL43-WT and pCMV(f)UL43-Y144 plasmids were transfected using Lipofectamine 2000 (Invitrogen, Carlsbad, CA, USA) according to manufacturer-specified guidelines. Additionally, Mock, UL43-WT and UL43-Y144A cells were cultured. The transfected cells were washed with PBS and then incubated with a resistant medium containing 10% FBS at 37 °C.

### 2.7. Endocytosis Assay

The transfected cells were washed with cold PBS and then incubated with a medium containing 10% FBS at 37 °C. At given times during the 37 °C incubation (0 h, 6 h or 12 h), the cells were fixed and permeabilized in 4% paraformaldehyde containing 0.2% Triton X-100. The cells were then incubated with anti-FLAG mouse monoclonal antibody (1:1000, Beyotime, Shanghai, China) for overnight, then incubated with goat anti-mouse IgG-RBITC (1:1000, Ricky, Shanghai, China) at 37 °C for 1 h. The co-localization of mitochondria and FLAG at 12 h post-transfection is based on this method, and incubated Mito-Tracker Green (Invitrogen, Carlsbad, CA, USA) was used to incubate the cells at 37 °C for 30 min in the dark according to the manufacturer ’s specified guidelines. The cells were washed twice with a fresh cell culture medium and then added to a fresh cell culture medium at 37 °C. The mitochondria were observed by LSM880 laser confocal microscopy (Carl Zeiss Inc., Oberkocken, Germany).

### 2.8. Analysis of Glucose Metabolism Level by UL43 Protein

In order to evaluate whether UL43 protein affects the aerobic oxidation of glucose, UL43-WT, UL43-Y144A and Mock cells were cultured in 6-well plates, and their supernatant was collected at 0, 4, 8, 16 and 24 h after transfection. In addition, the concentrations of glucose, pyruvate and lactate were measured using a Glucose Assay Kit, Pyruvate Assay Kit and Lactate Assay Kit, respectively (Biovision, Milpitas, CA, USA). Calculation of specific rates of glucose uptake was performed as described previously [22]. The levels of ATP in our cells were measured by the luciferin-luciferase method [23]. The enzyme activity of 6-phosphofructo-1-kinase, pyruvate kinase and lactate dehydrogenase was measured by the corresponding enzyme activity detection kit (Biovision, Milpitas, CA, USA).

### 2.9. Western Blotting

(a) Cells were harvested 24 h after HSV-1 or HSV-1 △UL43 infection and lysed with two folds dilutions of NP-40 lysis buffer (Sigma-Aldrich, St. Louis, MO, USA). Cell lysates were probed with antibodies of HSV-1 viral glycoprotein, and the VP5 signals were used as loading controls. (b) The expression of UL43-FLAG in mitochondria and other cell components of UL43-WT, UL43-Y144A and Mock cells was detected within 0, 6 and 12 h of transfection. We isolated and purified mitochondria from HUVEC cells according to the manufacturer’s protocol of the Cell Mitochondria Isolation Kit (Beyotime, Shanghai, China) and obtained mitochondrial proteins and residual cytoplasmic proteins after isolation. (c) UL43 binding protein in mitochondria of UL43-WT, UL43-Y144A and Mock cells were detected after transfection at 12 h; anti-phosphorylated monoclonal antibodies (Absin, Shanghai, China) were used to detect whether BART and ANT1 were phosphorylated. The collected samples were separated by the use SDS-PAGE (Tris-HEPES-SDS gradient 4 to 20% gels) followed by electrophoretic transfer to nitrocellulose membrane (Millipore, Billerica, MA, USA). The following mouse monoclonal primary antibodies were used: anti-gB (1:1000, Invitrogen, Carlsbad, CA, USA); anti-gD (1:1000, Invitrogen, Carlsbad, CA, USA); anti-gH (1:1000, Invitrogen, Carlsbad, CA, USA); anti-gL (1:1000, Invitrogen, Carlsbad, CA, USA); anti-VP5 (1:1000, Abcam, Cambridge, MA, USA); anti-Complex-I (1:1000, Invitrogen, Carlsbad, CA, USA); anti-COX-I (1:1000, Invitrogen, Carlsbad, CA, USA); anti-ARL2 (1:1000, Abcam, Cambridge, MA, USA); anti-BART (1:1000, Abcam, Cambridge, MA, USA); anti-phosphorylation of BART (1:1000,Absin, Shanghai, China); anti-phosphorylation of ANT1(1:1000, Absin, Shanghai, China); anti-β-Tubulin(1:1000, Abcam, Cambridge, MA, USA) and anti-VDAC1(1:1000, Abcam, Cambridge, MA, USA). The secondary antibody used was HRP-conjugated goat anti-mouse IgG (H + P, 1:5000, Abcam, Cambridge, MA, USA). Luminescence was assessed with an ECL developer, followed by imaging with an ultrasensitive Berthold Lumat LB9507 luminescence tube (Berthold Technologies, Bad Wildbad, Germany).

### 2.10. Measurement of Mitochondrial Membrane Potential

To measure mitochondrial membrane potential (MMP), 1 × 10^5^/mL cells on 6-well glass substrates (NEST, Wuxi, Jiangsu, China) were incubated with the following probes at 37 °C for 24 h: according to the reagent instructions, JC-10 dye 1:1000 was diluted with dye buffer, and 500μL dye solution was added to each well and incubated at 37 ℃ for 30 min. The fluorescence value was detected at 488/594 nm using LSM880 laser confocal microscopy.

### 2.11. Co-Immunoprecipitation

UL43-WT, UL43-Y144A and Mock cells were co-transfected with or without ARL2-siRNA. After 48 h of transfection, the cells were harvested, washed with PBS, and lysed in 500 μL NP40 lysis buffer (50 mM Tris-HCl, pH 8.0, 120 mM NaCl, 0.5% NP40, 1 mM PMSF). Mitochondrial proteins were extracted and incubated with primary antibodies (anti-FLAG, or ARL2 or lgG) at 4℃ overnight. According to the manufacturer ’s instructions (Invitrogen, Carlsbad, CA, USA), the protein G agarose beads were fully mixed with the antibody at 4 °C for 4 h. After immunoprecipitation, the beads were washed with a 1× phosphate-buffered saline (PBS) 3 times. The protein was eluted from the magnetic beads in 40 μL elution buffer and used for immunoblotting analysis. The sample buffer containing 5% β-mercaptothion was added to the sample and heated at 55 °C for 15 min, followed by the use of Western blotting to detect the binding protein.

### 2.12. MALDI-TOF-MS Mass Spectrometry

The position and molecular weight of the bands in the two gels were compared and grayscale analysis was carried out with Image J software (v1.51j8, Wayne Rasband National Institutes of Health, Bethesda, MD, USA). The samples with statistically significant differences (*p* < 0.05) were selected for MS mass spectrometry analysis. SDS-PAGE electrophoresis was performed with a 1.5 mg protein sample according to the above Western blot steps. The gel was stained with a Coomassie brilliant blue solution (containing 0.12% Coomassie Brilliant Blue G-250, 20% ethanol, 10% phosphoric acid, 10% ammonium sulfate). After staining, two different protein bands were cut. Each gel was digested with trypsin (Promega Corp, Madison, WI, USA) overnight at 37 ℃ for MALDI-TOF-MS mass spectrometry.

A MALDI-TOF/TOF mass spectrometer (AB SCIEX 5800, Carlsbad, CA, USA) was used for protein identification. A total of 0.6μL of the protein sample was treated with 1μL of 10 mg/mL α-cyano-4-hydroxycinnamic acid (CHCA) at 0.1% TFA and 50% acetonitrile (ACN), before being directly crystallized onto the target and vacuum-dried for detection. The equipment is set in linear mode, ion acceleration voltage set at 20 kV, N2 laser wavelength at 337 mm, pulse width at 3 ns, ion delay extraction at 500 ns, vacuum 5 × 10^5^ at Pa, and the spectrum is externally calibrated.

### 2.13. Measurement of mPTP Opening

A mitochondrial permeability transition pore (mPTP) detection kit (Beyotime, Shanghai, China) was used to detect the opening degree of mPTP in each group of cells, reflecting the concentration of calcium ions in mitochondria. Cells in each group were seeded in a confocal hole plate (NEST, Wuxi, Jiangsu, China), washed twice with PBS, for 1 min each time. 1 ml Calcein AM staining solution was added to each hole, and incubated in a 5% CO_2_ incubator at 37 °C for 30 min in the dark. After staining and incubation, the preheated culture medium at 37 °C was replaced, and then incubated at 37 °C for 30 min again. The laser confocal microscope was excited at a 488 nm wavelength. Other related treatment reagents were used to set blank and control groups: quenching working solution (Calcein AM containing 10% CoCl_2_), calcium overload solution (Calcein AM containing 10% CoCl_2_ and 5% lonomycin), and ARL2-siRNA treatment.

### 2.14. Statistical Analysis

All data are expressed as the arithmetic mean ± standard deviation (SD, n ≥ 3). Data were processed with GraphPad Prism 5.0 (San Diego, CA, USA) using one-way ANOVA, wherever appropriate. *p* < 0.05 was considered statistically significant.

## 3. Results

### 3.1. Deletion of the UL43 Protein-Coding Sequence Demonstrated a Minor Effect on Virus Production and Virion Entry

**Figure 1:**
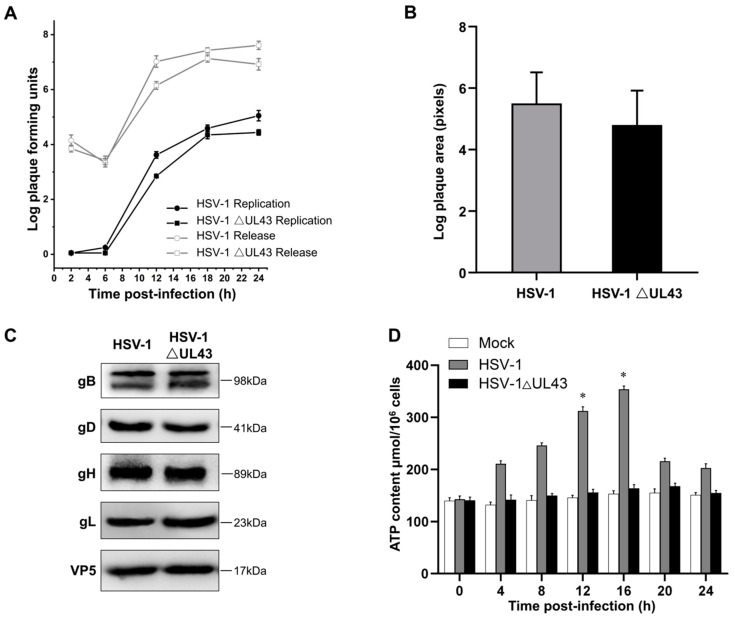
Growth and spread of viruses and viral glycoprotein expression levels in infected cells. (**A**) Single-step growth and supernatant virus curves for HSV-1 and HSV-1 △UL43 on HUVEC cells. (**B**) Sizes of plaques formed by HSV-1 and HSV-1 △UL43 on HUVEC cells. Data from one of three representative experiments are shown. (**C**) Western blot analysis of viral glycoprotein expression levels in infected cells. (**D**) ATP content assay of Mock, HSV-1 and HSV-1 △UL43. *: *p* < 0.05 vs. other groups.

It has been reported that the UL43 protein is not required for viral replication in cell culture [24], which correlates with our findings that single-step growth kinetics and plaque size of HSV-1 △UL43 were only marginally impaired compared to HSV-1. Here, we directly detected single-step growth kinetics and plaque areas on HEK293T cells and HUVEC cells (Figure 1A,B). Although HSV-1 △UL43 viral titers were similar to HSV-1, plaque size was reduced by approximately 10% (*p* < 0.05) on HUVEC cells. To evaluate the function of the UL43 protein in virus infection, the fusion of the viral glycoproteins gB, gD, gH and gL in infected HUVEC cells were analyzed by Western blotting. As shown in Figure 1C, the expression levels of glycoproteins gB, gD, gH and gL were highly similar between the HSV-1 △UL43 and HSV-1 viruses, indicating that alack of UL43 did not affect the fusion of viral glycoproteins.

We found that, after the HSV-1 infection of HCVEC cells, ATP levels began to change at 4 h and continued to increase, before they began to decrease at 16 h. Compared with the Mock group, the ATP level of HSV-1 △UL43 only slightly changed (Figure 1D). This indicates that UL43 protein significantly affects ATP energy levels in cells.

### 3.2. Microarray Data Analysis to Identify Genes Important for Viral Host Interactions

**Figure 2:**
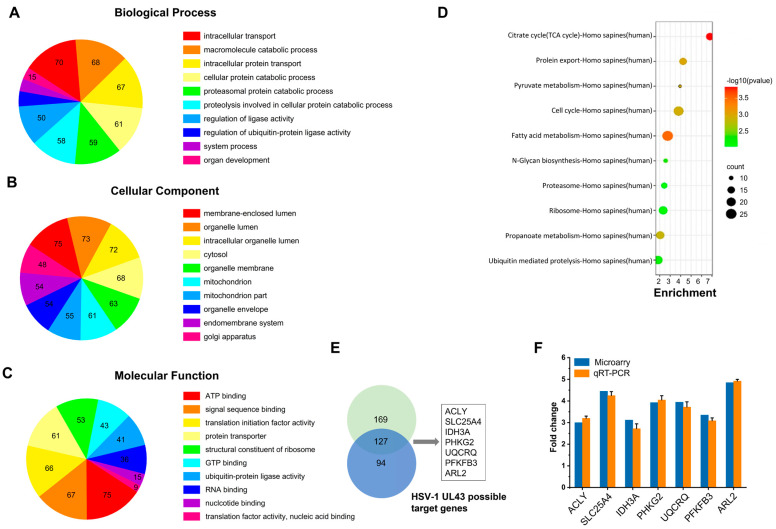
Microarray data analysis and validation of array data by quantitative real-time PCR. GO enrichment analysis of DEG: (**A**) biological process, (**B**) cellular component, (**C**) molecular function. (**D**) KEGG Pathway analysis of DEG showing the top ten enrichment scores of pathways. (**E**) Venn diagram of the significantly regulated DEG in multiples over two folds changes. (**F**) DEG were validated by qRT-PCR for microarray fold changes. Fold changes were determined by calculating the ratio of the mean expression values from the control and samples.

To identify cellular genes whose expression level change during HSV-1 infection, HUVEC cells were infected with HSV-1 and HSV-1 △UL43 mutants which had been successfully constructed. Compared with the HSV-1 group, more down-regulated genes were observed in the HSV-1 △UL43 group (Figure 2A). In this Gene Ontology annotation of cellular component and molecular function, most differentially expressed genes (DEG) belonged to the categories of response to mitochondrion, organelle membrane, organelle lumen (Figure 2B) and ATP binding, signal sequence binding (Figure 2C), respectively. Furthermore, DEG mainly affected TCA cycle, protein export, pyruvate metabolism (Figure 2D). We concluded that most of the DEG may be involved in cellular energy-yielding metabolic pathways.

Most of the known DEG may possess more interactions with other proteins. This was determined firstly through the network-based analysis of topological features, using Endeavour and ToppGene for the functional consistency analysis of related genes. Then, this was determined through the screening of (DEG) in multiples of 2 times or more than 2 times, after which we selected seven genes with each of the two methods (Figure 2E). Subsequently, seven commonly prioritized genes were exacted to improve the accuracy, after which they were molecularly annotated (Table S2).

### 3.3. Verification of Microarray Data for Selected Genes by qRT-PCR

To evaluate the reliability of the expression changes detected by the microarray analysis, we used qRT-PCR to analyze seven selected genes. The qRT-PCR results further confirmed the findings from the microarray analysis. In addition, seven selected genes represented the entire range of expression changes of about three times more (*p* < 0.05) (Figure 2F and Figure S1). The results showed that the detected gene expression was basically consistent with the microarray data.

### 3.4. Internalization and Localization of HSV-1 UL43 Protein to Mitochondria in Transfected HUVEC Cells

**Figure 3:**
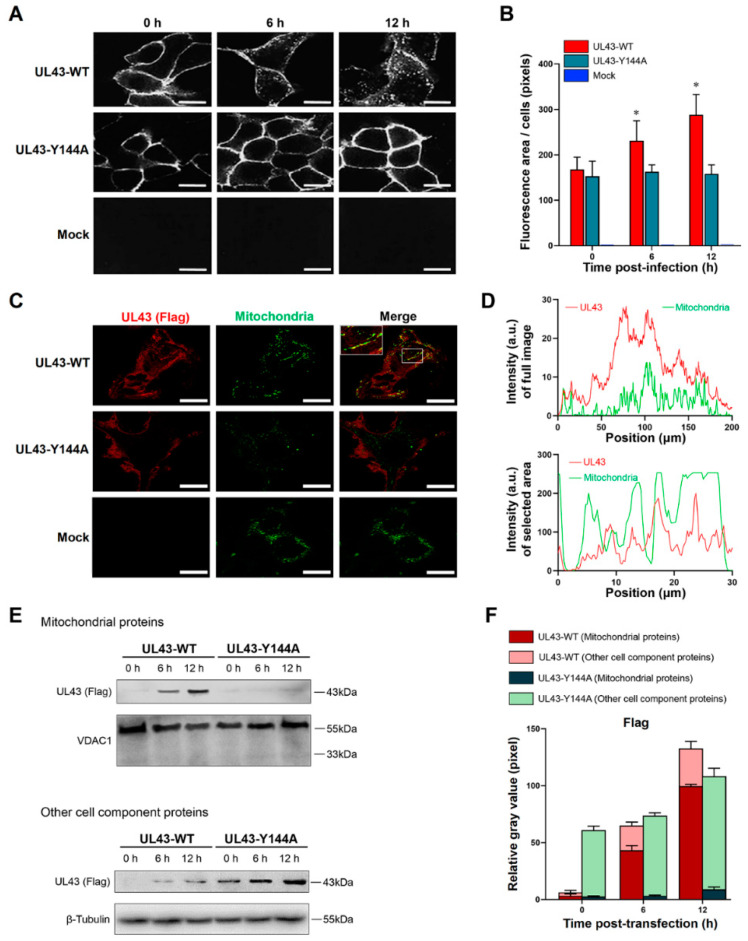
Internalization and localization of the HSV-1 UL43 protein to mitochondria. (**A**) Fluorescent image of UL43 protein internalization at 0, 6 and 12 h after transfection. (**B**) Quantitative analysis of average fluorescence areas of cells, *: *p*< 0.05 vs. other groups. (**C**) Co-fluorescent image of UL43 protein and mitochondria at 12 h after transfection. UL43-FLAG protein (red), mitochondria (green). (**D**). Fluorescence intensity analysis of UL-WT, both full-size image and mitochondrial region. (**E**) Western blot analysis of UL43-FLAG expression in mitochondria and other cell components, at 0, 6 and 12 h after transfection. (**F**) Relative quantitative analysis of UL43 FLAGs expression in mitochondria and other cell components. Scale bar = 5 μm. Groups: UL43-WT (pCMV(f)UL43-WT plasmid), UL43-Y144A (pCMV(f)UL43-Y144A plasmid), Mock (empty plasmid).

To determine whether the YPLF motif in the cytosolic domain of HSV-1 UL43 protein directs UL43 protein internalization, HUVEC cells which express wild-type UL43 (UL43-WT) were examined, as were those displaying the derivative site-specific mutation UL43-Y144A. Immunofluorescence analysis showed that the cell periphery of UL43-WT was circularly stained at 0 h after transfection. At 6 h, a small part of the cell surface UL43 protein was internalized; up to 12 h, cytoplasmic vesicles were strongly stained (Figure 3A), indicating that most UL43 protein on the cell surface was internalized. However, analysis of substitution mutation UL43-Y144A declared that they were impaired in internalization. The ringlike pattern observed for UL43-Y144A at 0 h was not changed appreciably at 6 h and 12 h. UL43-Y144Amutant was poorly internalized compared with UL43-WT (Figure 3B), which suggested the importance of the YPLF motif in directing UL43 trafficking in the endocytic pathway. To determine the intracellular localization of UL43 protein, confocal imaging analysis showed that UL43 protein colocalized with mitochondria in HUVEC cells transfected with UL43-WT (Figure 3C,D). In addition, UL43 protein was detected in mitochondria of HUVEC cells transfected with UL43-WT by Western blot at 6 h and 12 h and increased with time, while UL43-Y144A was not detected (Figure 3E,F). After the separation of mitochondria, we detected the cell protein, and it was found that the expression of UL43 protein transfected with UL43-Y144A gradually increased with time, but gradually decreased in UL43-WT. These results illustrated that UL43 protein endocytosis can rely on the YPLF motif and locates to mitochondria to affect host cell metabolism.

### 3.5. UL43 Transfection Induces Aerobic Oxidation of Glucose

**Figure 4:**
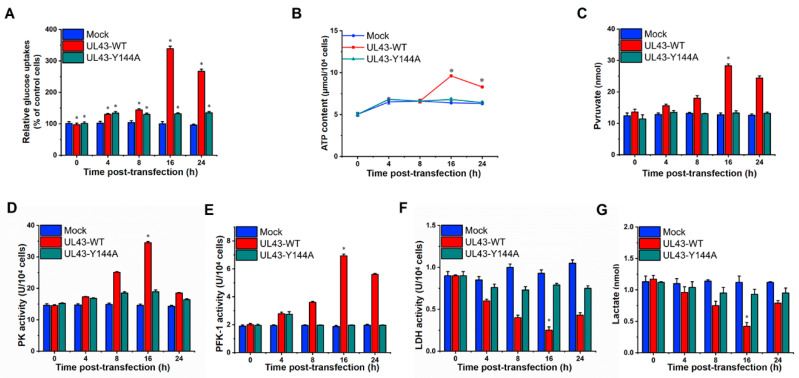
UL43-transfection induces aerobic oxiation of glucose. The oxidative metabolism levels of UL43-WT, UL43-Y144A and Mock cells were detected at 0, 4, 8, 16 and 24 h. (**A**) Relative glucose uptake analysis. (**B**,**C**) Analysis of ATP content and pyruvate level. (**D**–**F**) Enzyme activity detection of PK, PFK-1, LDH. (**G**) Analysis of lactate level. *: *p*< 0.05 vs. other groups.

Glycolysis and the TCA cycle form the backbone of central carbon metabolism in mammalian cells. Through these two pathways, glucose is either oxidized to produce energy in the form of NADH and ATP, or converted to precursors of amino acids, lipids and nucleotides. Therefore, we decided to further investigate the mechanism of UL43 protein-induced activation of aerobic oxidation of glucose. Interestingly, the glucose uptake of HUVEC cells transfected with UL43-WT was moderately enhanced at 8 h after transfection, and more significant changes were observed at 16 h after transfection, as measured by the decreased glucose concentration in the supernatant of HUVEC cells (*p* < 0.05) (Figure 4A). Conversely, glucose uptake seemingly indicated no significant difference in UL43-Y144A-transfected cells, as compared with the Mock-transfected cells. Cellular ATP levels were affected by aerobic oxidation of glucose and may regulate glycolytic flow, and therefore we measured the ATP levels of cells. It was found that UL43-WT-transfected cells had approximately two-fold higher levels of ATP than Mock and UL43-Y144A transfected cells at 16 h post-transfection and slightly increased at 4 h (Figure 4B).

Thus, we next investigated whether UL43 regulated the key rate-limiting enzymes of aerobic oxidation of glucose. The key rate-limiting regulatory enzymes of the glycolytic pathway are PFK-1 and pyruvate kinase (PK). We found that UL43-WT- transfection robustly increased PFK-1 and PK activity and concentrations of pyruvate at 16 h post-transfection, but only slightly increased those in UL43-Y144A-transfected cells (Figure 4C–E). In parallel, we also analyzed other key regulators of aerobic oxidation of glucose and found that lactate dehydrogenase (LDH) was influenced by UL43-WT-transfection, which suggested that UL43 remarkably decreased LDH activity and concentrations of lactate at 16 h post-transfection, but only slightly decreased in cells transfected with Mock and UL43-Y144A (Figure 4F,G). In conclusion, those results could be explained by enhanced glycolytic catabolic flux, which led to an increase ib the intracellular ATP content.

### 3.6. UL43 Protein Facilitates the Process of Oxidative Phosphorylation

**Figure 5:**
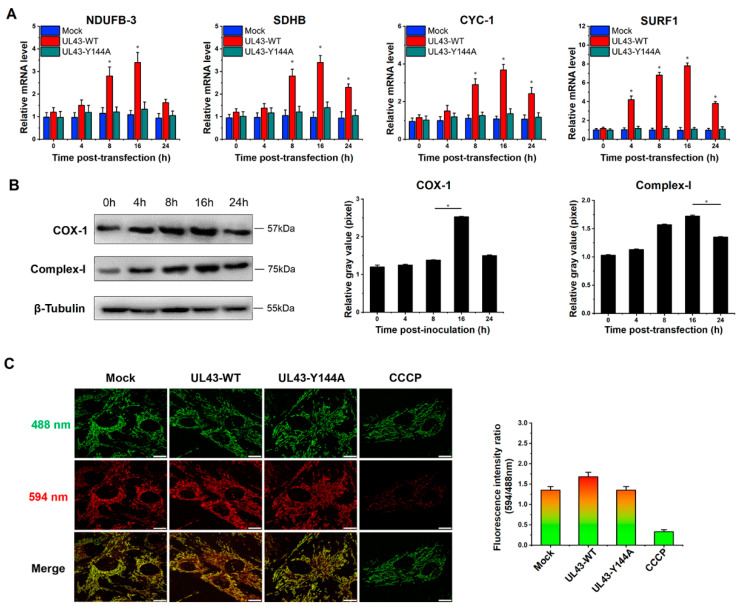
UL43 transfection results in an abundance of mRNA, corresponding to several OXPHOS-related enzymes. UL43-WT, UL43-Y144A and Mock cells were detected at 0, 4, 8, 16 and 24 h. (**A**) Quantitative analysis of the mRNA expression level of NDUFB3, SDHB, CYC1 and SURF1 by qRT PCR. (**B**) Western blot analysis and grayscale quantification statistics are used to analyze the expression level of Complex-I and COX-I, *: *p* < 0.05. (**C**) Fluorescence image of mitochondrial membrane potential analysis and quantitative analysis of fluorescence ratio.

To further analyze how UL43 protein affected the intracellular energy system, we tested the activity of electron transport chains in mitochondria. NADH dehydrogenase (ubiquinone) 1 beta subcomplex subunit 3 (NDUFB3), succinate dehydrogenase subunit B (SDHB), cytochrome c-1 (CYC1) and surfeit locus protein 1 (SURF1) were subunits of four membrane-bound complexes, including Complex I, II, III and IV. Therefore, we detected the mRNA expression of the four genes via the qRT-PCR technique, and the result displayed that the mRNA levels were robustly increased at 16 h post-transfection with UL43-WT. In contrast, there was almost no change in the cells transfected with UL43-Y144A compared with the Mock-transfected cells (Figure 5A). Moreover, Cytochrome c oxidase subunit I (COX-I) was one of three mitochondrial DNA (mtDNA)-encoded subunits of respiratory complex IV. We further analyzed the protein expression of Complex-I and COX-I by Western blotting. In truth, an increase in the protein expression starting at 4 h post-transfection and more pronounced expression of Complex-I and COX-I we both observed at 16 h (Figure 5B). Overall, UL43 protein could activate electron transport chain activities through the protein expression increasing, such as Complex I, II, III and IV.

We also examined mitochondrial membrane potential (MMP) in UL43-transfected cells, an important parameter of cellular metabolism and mitochondrial energy status. Therefore, we performed a state experiment of MMP 24 h after transfection. Compared with UL43-Y144A-transfected cells, UL43-WT-transfected cells showed higher red:green fluorescence intensity (Figure 5C), indicating that UL43 leads to a continuous increase in MMP and promotes the mitochondrial electron transport activity produced by ATP in the electron transport chain.

### 3.7. UL43 Protein Binds to ARL2

**Figure 6:**
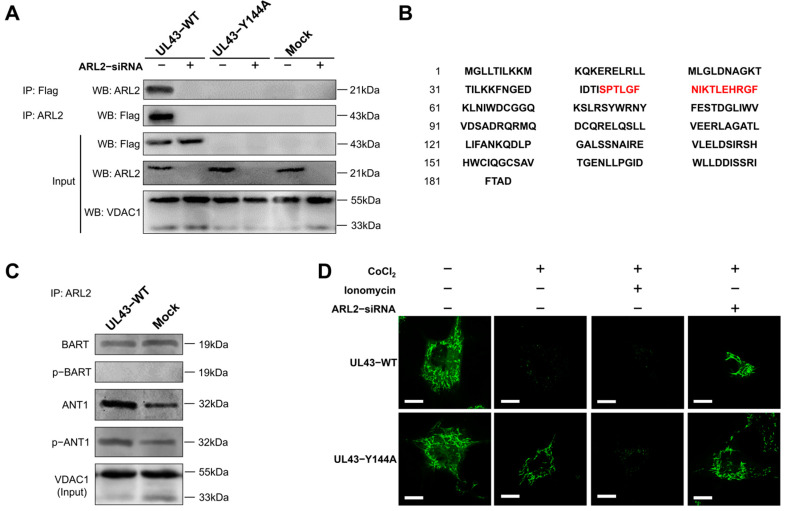
UL43 protein binding to ARL2 and phosphorylation of ANT1. (**A**) Co--immunoprecipitation assay in mitochondrial proteins of UL43--WT, UL43--Y144A, Mock, performed with co-transfect of ARL2-siRNA. (**B**) The peptide sequence is detected by mass spectrometry, and the ARL2-specific binding sequence is marked in red. (**C**) Co-immunoprecipitation assay in mitochondrial proteins of UL43-WT and Mock, detecting the phosphorylation level of BART and ANT1. (**D**) Fluorescent image of mitochondrial membrane pores opening test, UL43-WT and UL43-Y144A were treated with CoCl_2_, ionomycin and ARL2-siRNA. Scale bar = 5 μm. The data shown in each panel are representative of three independent experiments.

To clarify the function(s) of UL43 in host cell metabolism, we tried to identify the host mitochondrial proteins that interact with the UL43 protein. Seven differentially expressed genes (ACLY, SLC25A5, IDH3A, PHKG2, UQCRQ, PFKFB3, ARL2) through microarray data analysis and subunits of four membrane-bound complexes (NDUFB3, SDHB, CYC1, SURF1) were associated with the UL43 protein in mammalian cells. To verify whether UL43 in fact associates with these genes, we adopted the co-immunoprecipitation. In the mitochondria of transfected HUVEC cells, UL43-Y144A-expressed protein did not demonstrate binding to ARL2, while UL43 protein in UL43-WT of HUVEC cells could combine with ARL2, which is related to the inability of UL43-Y144A to locate mitochondria. When we used ARL2 siRNA with knockdown efficiency of 82.36 ± 5.66% to reduce ARL2 expression, this binding was significantly weakened, as shown in Figure 6A. The peptide sequence of the UL43-WT targeted protein is S-P-T-L-G-F-N-I-K-T-L-E-H-R-G-F (Figure 6B). Consistent with the amino acid sequence of ARL2 (Supplementary Table S3) shown in the Uniort database, these results indicate that UL43 binds to ARL2.

### 3.8. UL43 Transfection Makes ARL2 Phosphorylate ANT1

Because it has been reported that ARL2, BART and ANT1 proteins exist in a complex binding state [25], we examined the effect of UL43 on BART and ANT1 through ARL2. Through co-immunoprecipitation, we verified that ARL2, BART and ANT1 form an interactive complex, and that the phosphorylation of ANT1 is significantly enhanced when transfected with UL43-WT (Figure 6C). These results indicate that the coupling of UL43 protein with ARL2 may rapidly activate ANT1 phosphorylation. The results demonstrated that UL43 was localized to the mitochondrial membrane and bound to ARL2, which activated ANT1 phosphorylation and affected ATP production.

Mitochondrial permeability transition pores (mPTP) form a group of protein complexes between the inner and outer membranes of mitochondria. They constitute a voltage-dependent anion channel of the outer membrane voltage-dependent anion channel (VDAC),the inner membrane adenine nucleotide translocator protein (ANT) and the cyclophilin D voltage-dependent anion channel. The weaker the green fluorescence detected, the higher the openness of mPTP. UL43-WT cells without ARL2-siRNA treatment can quench the fluorescence in mitochondria under the action of CoCl_2_, which indicates a higher level (or frequency) of mPTP channel opening than UL43-Y144A cells. When the cells were treated with ARL2-siRNA, there was no significant difference in mitochondrial fluorescence intensity between UL43-WT and UL43-Y144A, suggesting that UL43 could not phosphorylate ANT1 when it lacked its substrate ARL2 (Figure 6D). This indicates that the UL43 protein is coupled with ARL2 to phosphorylate ANT1, resulting in increased opening of mPTP.

## 4. Discussion

As viruses are parasitic to cells, matter and energy from host cells are indispensable for the replication and assembly of virus reproduction. The structure of HSV-1 genome arranges as follows: TRL-UL-IRL-IRS-US-TRS. On one hand, it seems that many ULs protein-like virus domains are targets to mitochondria and interact with mitochondrial proteins. For example, UL12.5 proteins degrade mitochondrial DNA [15], and UL7 and UL16 also target mitochondria [14,26,27]. On the other hand, US3 and UL13 encode protein kinases that participate in the process of protein phosphorylation [28,29]. Our study confirmed that HSV-1 UL43 protein is a target for mitochondria and affects the channel transport function of mitochondrial ATP production.

UL43 is a non-essential protein for viral replication. We demonstrated that HSV-1 virus, lacking the UL43 gene, exerts no impact on its membrane fusion. The UL43 deletion virus also maintains viral infection activity, but the ATP metabolism level of the host was affected significantly. Bioinformation analysis of microarrays and qRT-PCR confirmed that UL43 would mainly affect the energy metabolism of host cells, including ACLY, SLC25A4 (encoding ANT1), IDH3A, PHKG2, UQCRQ, PFKFB3, and ARL2.

UL43 protein is a highly hydrophobic, seven-layer transmembrane protein. Many virus-encoded membrane proteins, including HSV-1 gB, gD, and gE envelope proteins contain endocytosis motifs that undergo endocytosis in transient expression systems, which are mediated by a specific amino acid sequence located in the cytoplasmic domain [30]. The endocytosis signals characterized best are the YXXΦ motif (where Y is tyrosine, X is any amino acid, Φ is any large hydrophobic amino acid) and the LL (bisleucine) motif [31]. These motifs initiate endocytosis by establishing the interaction of AP-2 ligand complexes associated with grid proteins, which is the first step in the formation of grid protein-coated vesicles [32,33]. Our previous studies have shown that UL43 has a YPLF amino acid motif (aa144-147) and cannot form complexes with clathrinid-associated AP-2 ligands [18]. Therefore, little is known about the function of UL43 on YPLF motif and whether it belongs to the YXXΦ motif. In this study, we found that mutations in tyrosine residues in the YPLF motif significantly reduced the endocytosis efficiency of the UL43 protein, suggesting that the YPLF motif belongs to the YXXΦ endocytosis motif. This proves that the endocytosis of UL43 is mediated by the YPLF motif, and that this endocytosis is the basis for UL43 to exert biological functions in host cells.

With regard to the LL motif in UL43 protein, compared with YXXΦ motif, LL endocytosis signal is generally considered to be a relatively weak endocytosis signal. The mutation of tyrosine residues only destroys the YXXΦ endocytosis motif in the YPLF sequence, but not the LL motif. However, the function of the LL endocytosis signal is still unknown. More research is needed to clarify the problem.

In this study, we transfected UL43-Y144A and UL43-WT with mutations in the tyrosine residue of the YPLF motif and found that UL43 was internalized and localized to the mitochondria and binds to the ARL2 protein, constituting the ANT1 phosphorylation chain of action. This chain of action significantly affects ATP levels in host cells. ATP levels in cells transfected with UL43-WT after 16 h are about twice as high as those transfected with UL43-Y144A, promoting the activity of key rate-limiting enzymes in glucose aerobic oxidation. By qRT-PCR and Western blot, it was found that the activity of electron transport chain in mitochondria of cells transfected with UL43-WT was enhanced and the membrane potential was increased. This indicates that the internalization and localization of UL43 in mitochondria significantly increases the oxidative phosphorylation of the host and increases ATP production, performing the biological function of transmembrane signaling and affecting host energy metabolism. Co-immunoprecipitation and proteomic analysis showed that UL43 protein bound to ARL2. Phosphorylation of ANT1 protein was detected in mitochondria isolated from HUVEC cells, and significant differences in ANT1 expression were also observed in microarray data analysis. ANT1 is a member of the mitochondrial inner membrane permeability replacement pore complex, and there are multiple phosphorylation sites. The increase in ANT1 phosphorylation level will affect the opening of intracellular permeability replacement pores to change the ratio of ATP/ADP [34]. Based on these facts, the molecular mechanism of UL43 involved in the control of mitochondrial ATP production was elucidated. That is, UL43 activated the cellular ATP energy generation system by triggering ANT1 phosphorylation via the action of coupling with the ARL2(GTP)-BART complex to affect the openness of mPTP.

α-, β- and γ-herpesviruses encode homologues of human chemokine receptors, which are called viral G protein-coupled receptor (vGPCR) proteins, and most vGPCRs bind to chemokines [35,36]. G protein-coupled receptor (GPCR) and its G protein usually activate cell phosphorylation pathways to affect cell function. However, α-herpesviruses does not produce vGPCR. Based on our study, we speculate that the UL43 protein is a highly hydrophobic multi-transmembrane protein similar to GPCR. However, unlike vGPCR encoded by β and γ herpesviruses, the UL43 protein can directly bind to small G proteins and initiate phosphorylation of downstream target molecules, which may represent the discovery of a new mechanism of vGPCR viral proteins and molecules.

As a small G protein, ARL2 acts as a molecular switch in the mitochondria inside the outer mitochondrial membrane. ARL2 is activated by binding to GTP, thus promoting the binding of ARL2 to ARL2 effect combiners (BART or ARL2BP) [37]. ANT1 is the main binding chaperone of the ARL2 (GTP)-BART complex [25,38]. ANT protein is a rich and indispensable component of the mitochondrial inner membrane. It dimerizes on the inner membrane and forms mPTP of ATP and ADP exchange channels, thereby controlling ATP levels in the cytoplasm [39,40]. Recent studies have reported that HSV-1 protein UL16 binds to ANT to promote mitochondrial energy metabolism [27]. ANT, which located in mitochondrial mPTP, may be the main way for the virus to quickly obtain ATP from host cells.

In summary, this study shows that UL43 protein, as a non-essential protein, has no significant effect on HSV-1 replication, but can enhance the energy metabolism pathway of host cells and has a broader significance for HSV-1 virus replication. It is found that the UL43 protein is internalized to the mitochondrial membrane and coupled to the small G protein ARL2. ARL2 is activated after binding to GTPase, and the ARL2 (GTP)-BART complex can bind to ANT1, trigger the phosphorylation of ANT1, and regulate the opening activity of mPTP, thereby changing the transport rate of ATP from one based on mitochondria to one based on the cytoplasm, and the transport rate of ADP from one based cytoplasm to one based on mitochondria. The interaction between UL43 protein of HSV-1 and ATP-related signaling pathways in mammalian host mitochondria was elucidated. This study first reported the mechanism by which UL43 affects host ATP production, providing a new way to understand the molecular mechanism of interaction between viral genes and host cells. In addition, the activation of mitochondrial ATP by the UL43 protein may play an important role in energy metabolism disorders (such as cell senescence) in mammalian cells.

## Data Availability

The original contributions presented in the study are included in the article/Supplementary Material. Further inquiries can be directed to the corresponding authors.

## Supplementary Materials

The following supporting information can be downloaded at: https://www.mdpi.com/article/10.3390/cells11223594/s1, Figure S1: DEG expression level of HSV-1 and HSV-1 △UL43 tested by qRT-PCR; Table S1: The primers of selected genes for quantitative real-time PCR; Table S2: Functions of candidate genes in human; Table S3: Peptide Mass of ARL2.
