# Supplementary material for: Herpes Simplex Type 1 UL43 Multiple Membrane-Spanning Protein Increases Energy Metabolism in Host Cells through Interacting with ARL2
Source: Cells. 2022 Nov 14;11(22):3594. doi: 10.3390/cells11223594 (PMC9688820; doi:10.3390/cells11223594)
Supplement: Supplementary file 1 [file cells-11-03594-s001.zip › cells-2011596-supplementary.pdf]

Supplementary Materials for

‘Herpes simplex type 1 UL43 multiple membrane-spanning protein increases energy metabolismin in host cells through interacting with ARL2’

Jianshan Deng [1,†], Zhiying Zhong [1,†], Chengxu Geng [1,†], Zhenning Dai [2,3], Weihan Zheng [2], Ziyue Li [2], Zi Yan [2], Jiaxin Yang [4], Wenfeng Deng [5], Wei Tan [2,6,*], Hanxiao Sun [1,*] and Shiyu Li [1,2,4,*]

[1] Institute of Genomic Medicine, College of Pharmacy, Jinan University, Guangzhou 511436, China
[2] Guangdong Medical Innovation Platform for Translation of 3D Printing Application, The Third Affiliated Hospital of Southern Medical University, Southern Medical University, Guangzhou 510630, China
[3] Department of Stomatology, Guangdong Second Traditional Chinese Medicine Hospital, Guangzhou 510095, China
[4] Department of Anatomy, Guangdong Provincial Key Laboratory of Digital Medicine and Biomechanics, School of Basic Medical Sciences, Southern Medical University, Guangzhou 510515, China
[5] Guangzhou Women and Children’s Medical Center, Guangzhou Medical University, Guangzhou 510799, China
[6] Guangxi Key Laboratory of Birth Defects Research and Prevention, Nanning 530005, China
* Correspondence: author: tanwei@smu.edu.cn (W.T.); hanxiaosun718@jnu.edu.cn (H.S.); li594171833@smu.edu.cn (S.L.)
† These authors contributed equally to this work.

This PDF file includes:

Supplementary Table S1 to S3, Figure S1 and Western blots figures of the manuscript

Table S1. The primers of selected genes for quantitative real-time PCR

Gene title	Forward primer	Reverse primer	Size(bp)
ACLY	5’-GGTGGGGACAGGTAATCAGC-3’	5’-CATCGTCTCCTAGCCCACTG-3’	74
SLC25A4	5’-CCAAGTCAAGGGATGGGGTT-3’	5’-GCCCCACCTCTAAGAACGTC-3’	216
IDH3A	5’-CTCGCAGTGTATGGGCCG-3’	5’-TGCTGGTCTCTCTCTCCGAA-3’	497
PHKG2	5’-GCCAAACTGCCTTGTTGGAG-3’	5’-GGCTCAACCCTTACCTGGTT-3’	242
UQCRQ	5’-GTCTTTCTTTCGCGTGGTGC-3’	5’-GTGACCTCAGAGGAACATGC -3’	280
PFKFB3	5’-TGATTCGGCCACCTTGACTC-3’	5’-ACTTTGCCGCATAAGGCTCT-3’	208
ARL2	5’-AGCGTGATAGCCAACAGGA-3’	5’-GCAAGGAGCGGTTTATTTCA-3’	105
NDUFB3	5’-TTAGGGAGTTGGTGGGAGGC-3’	5’-GTTCTTCTGCCTTTGCGATCC-3’	284
SDHB	5’-GAGAGCGACCTCGGGGTTA-3’	5’-CTTTTCCCTCTCTGAGGCTCC-3’	149
CYC1	5’-ATGGTAAGAGGCCTCCAGTCT-3’	5’-AAGCCTCCTGAGACATGCAC-3’	143
SURF1	5’-ACTTCCGTACGTTGTGGACC-3’	5’-GTCATTTTTGCGTGGCCAGT-3’	240

Table S2. Functions of candidate genes in human

		Description		
**Gene ID**	**Symbol**	**Biological process**	**Cellular component**	**Molecular function**
47	ACLY	citrate metabolic process	mitochondrion	ATP binding
291	SLC25A4	Energy reserve metabolic process	positive regulation of cell proliferation	protein binding
3419	IDH3A	carbohydrate metabolic process	mitochondrial matrix	NAD binding
5261	PHKG2	carbohydrate metabolic process	phosphorylase kinase complex	ATP binding
27089	UQCRQ	cellular metabolic process	mitochondrial inner membrane	ubiquinol-cytochrome-c reductase activity
407183	PFKFB3	fructose metabolic process	cytoplasm	ATP binding
402	ARL2	small GTPase mediated signal transduction	mitochondrial intermembrane space	GTP binding

Gene products have several functions, which have many GO terms, but only one is included in the table.Biological process, cellular component, and molecular functions predicted by the GO annotation system and Uniprot database.

Figure S1. DEG expression level of HSV-1 and HSV-1 △UL43 tested by qRT-PCR

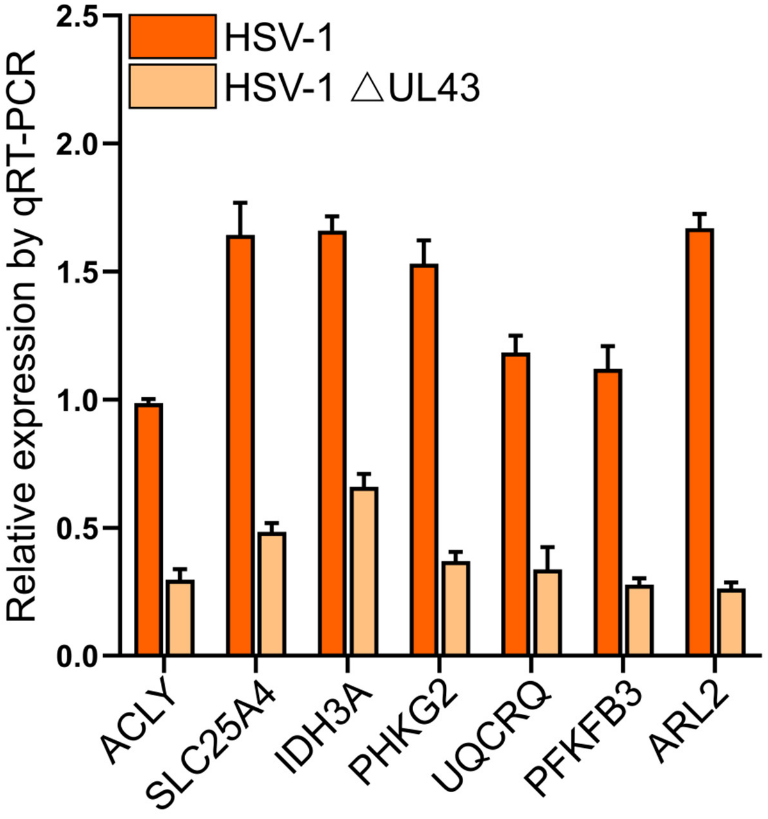

Table S3. Peptide Mass of ARL2

The entered protein is: Q19705

The selected enzyme is: Trypsin

Maximum number of missed cleavages (MC): 0

All cysteines in reduced form.

Methionines have not been oxidized.

Displaying peptides with a mass bigger than 500 Dalton.

Using monoisotopic masses of the occurring amino acid residues and giving peptide masses as [M+H]+ .

Chain ADP-ribosylation factor-like protein 2 at positions (Q19705) [Theoretical pI: 6.48 / Mw (average mass): 20868.13 / Mw (monoisotopic mass): 20854.91]

mass	position	peptide sequence
2305.2397	127-148	SDLPGAIDVNSIAQVLDLHS IK
2116.9821	79-96	NYFESTDALIWVVDSSDR
2051.0219	36-53	FLDEPTDTIEPTLGFDIK
1693.8036	165-179	LVQAMTWLCDDVGSR
1519.7539	59-71	DFQLNLWDVGGQK
1171.5234	154-164	IFSCCALSGDR
1171.5234	154-164	IFSCCALSGDR
1169.7252	115-126	LAGASLLVLANK
1126.6830	19-29	ILILGLDNAGK
1062.5499	99-107	LLQCSEELK
716.3937	109-114	LLGEER
694.3419	149-153	SHHWK
631.3562	54-58	TVHFK
620.3654	180-184	LFILD
593.3327	30-34	TTLMK
583.2875	75-78	SYWK

The data in this table on protein profiles come from the UniProtKB/Swiss-Prot database（https://web.expasy.org/cgi-bin/peptide_mass/peptide-mass.pl）
